# Inhibiting the P2X4 Receptor Suppresses Prostate Cancer Growth In Vitro and In Vivo, Suggesting a Potential Clinical Target

**DOI:** 10.3390/cells9112511

**Published:** 2020-11-20

**Authors:** Jiepei He, Yuhan Zhou, Hector M. Arredondo Carrera, Alexandria Sprules, Ramona Neagu, Sayyed Amin Zarkesh, Colby Eaton, Jian Luo, Alison Gartland, Ning Wang

**Affiliations:** 1 The Mellanby Centre for Bone Research, Department of Oncology and Metabolism, The University of Sheffield, Beech Hill Road, Sheffield S10 2RX, UK; jhe44@sheffield.ac.uk (J.H.); yzhou1107@gmail.com (Y.Z.); hmarredondocarrera1@sheffield.ac.uk (H.M.A.C.); arfsprules1@sheffield.ac.uk (A.S.); rneagu1@sheffield.ac.uk (R.N.); zarkeshamin@gmail.com (S.A.Z.); c.l.eaton@sheffield.ac.uk (C.E.); a.gartland@sheffield.ac.uk (A.G.); 2 Shanghai Key Laboratory of Regulatory Biology, Institute of Biomedical Sciences and School of Life Sciences, East China Normal University, Shanghai 200241, China; jluo@bio.ecnu.edu.cn

**Keywords:** purinergic signalling, P2X4 receptor, prostate cancer, ATP, antagonist, xenograft model, clinical datasets

## Abstract

Prostate cancer (PCa) is the most frequently diagnosed cancer in men, causing considerable morbidity and mortality. The P2X4 receptor (P2X4R) is the most ubiquitously expressed P2X receptor in mammals and is positively associated with tumorigenesis in many cancer types. However, its involvement in PCa progression is less understood. We hypothesized that P2X4R activity enhanced tumour formation by PCa cells. We showed that P2X4R was the most highly expressed, functional P2 receptor in these cells using quantitative reverse transcription PCR (RT-PCR) and a calcium influx assay. The effect of inhibiting P2X4R on PCa (PC3 and C4-2B4 cells) viability, proliferation, migration, invasion, and apoptosis were examined using the selective P2XR4 antagonists 5-BDBD and PSB-12062. The results demonstrated that inhibiting P2X4R impaired the growth and mobility of PCa cells but not apoptosis. In BALB/c immunocompromised nude mice inoculated with human PC3 cells subcutaneously, 5-BDBD showed anti-tumourigenic effects. Finally, a retrospective analysis of *P2RX4* expression in clinical datasets (GDS1439, GDS1746, and GDS3289) suggested that P2X4R was positively associated with PCa malignancy. These studies suggest that P2X4R has a role in enhancing PCa tumour formation and is a clinically targetable candidate for which inhibitors are already available and have the potential to suppress disease progression.

## 1. Introduction

Prostate cancer (PCa) is the second most frequently diagnosed cancer (more than 1.3 million new cases worldwide in 2018) and the fifth leading cause of cancer deaths (over 350,000 deaths annually) in men [1]. The life-time risk of developing PCa is >10% [2], while the incidence of PCa between the ages of 65 to 74 is as high as 35.3% [3]. The mortality rate also rises with age, and 55% of PCa deaths occur over the age of 65 [1]. With advances in treatments, patients with localized PCa have a very good prognosis (five-year survival rate >99%). However, metastatic PCa patients only have a five-year survival rate just above 30% [2]. More importantly, a trend towards an increased PCa incidence and a doubling of mortality is estimated worldwide up to the year 2040, particularly affecting developing countries in Africa, Latin America, the Caribbean, and Asia [1]. Effective treatment strategies, in particular for the suppression of disease progression for the majority of patients that present with clinically localised disease, as well as better diagnostic and prognostic biomarkers, are urgently needed.

P2X receptors are a group of ATP-gated nonselective ionotropic channels, currently with seven known mammalian members (P2X1–P2X7) [4,5]. The P2X4 receptor (P2X4R) was first cloned in the late 1990s [6,7] and is a typical P2X receptor, containing two transmembrane domains (TM1 and TM2), an intracellular domain with an N-terminus and C-terminus, and a large extracellular loop domain with three ATP binding sites [8]. P2X4R is one of the most sensitive and ubiquitously expressed P2X receptors [9,10]. P2X4R is prominently expressed in central and peripheral neurons, immune cells, and endothelial cells [11]. At the intracellular level, P2X4R localises on the plasma membrane, as well as in intracellular compartments such as lysosomes and vesicles [12]. This wide distribution indicates important regulatory roles of P2X4R in multiple biological and pathological processes, such as pain [13,14], central nervous system (CNS) disorders [11,15], inflammation [16], and cardiovascular diseases [17,18]. P2X4R are also expressed in many cancer types, including lung, colorectal, bladder cancer, leukaemia, brain tumour, and, most importantly to this study, PCa [19]. Functionally, most studies suggest that P2X4R positively associates with tumourigenesis [20,21,22]. However, whether and how P2X4R is involved in PCa progression remains to be elucidated. Limited evidence showed that the antidepressant paroxetine, a P2X4R inhibitor, induced [Ca^2+^]_i_ rises in human PCa cells [23], indicating a functional role of P2X4R in PCa progression [24]. More recently, P2X4R was shown to be involved in TGFβ-1 induced invasiveness and epithelial-to-mesenchymal transition (EMT) in PCa cells [25]. These studies suggested a complex link between P2X4R and PCa activities. Here, we comprehensively define the roles of P2X4R in PCa progression and test the hypothesis that P2X4R activity enhances tumour formation by PCa cells.

To achieve this objective, we have first examined the transcriptional expression of P2 receptors in several PCa cells lines and determined whether the expressed P2X4R are functional. PCa cell viability, proliferation, migration, invasion, and apoptosis were examined in PCa cells treated with the P2X4R selective antagonists 5-BDBD and PSB-12062. A xenograft murine model was further used to determine whether the blockade of P2X4R using 5-BDBD impaired the PCa tumour growth in vivo. Finally, using publicly available clinical datasets, the association between P2X4R expression and PCa disease progression was established.

## 2. Materials and Methods

### 2.1. Cell Culture

The PC3 prostate cancer cell line (Prostate Specific Antigen (PSA) non-expressing, androgen-independent) (ATCC, Manassas, VA, USA) was stably transfected with a firefly luciferase gene luc2 (pGL4.51 [luc2/CMV/Neo] vector, Promega, Madison, WI, USA) using a Gene PulserTM electroporator (Bio-Rad, CA, USA). LNCaP (PSA expressing, androgen-sensitive) were purchased from ATCC, and the LNCaP-derived C4-2B4 strain was supplied by the University of Bern (Bern, Switzerland). All cell lines were maintained in Dulbecco’s Modified Eagle’s Medium (Gibco, Thermo Fisher Scientific, Waltham, MA, USA), supplemented with antibiotics and foetal bovine serum (FBS) (Sigma Aldrich, St. Louis, MO, USA). All cell lines were genetically profiled by Stem Elite ID system (Promega, Madison, WI, USA), which confirmed their identity (18/18 STRs) and regularly screened for mycoplasma contamination.

### 2.2. Quantitative Real-Time PCR

Total RNA were extracted from PCa cell lines using the ReliaPrep™ RNA Cell Miniprep System (Promega). cDNA was synthesized using SuperScript™ III reverse transcriptase (Invitrogen, Life Technologies) with a 1:1 mix of Oligo(dT) 15 and Random primers (Promega). TaqMan Gene Expression Assays were then used to quantify the expression of P2 receptors with an Applied Biosystems 7900HT Real-Time PCR system (Applied Biosystems, Thermo Fisher Scientific) (50 °C, 2 min, 94.5 °C, 10 min, followed by 40 cycles of denaturation at 97.0 °C for 30 s and an extension at 59.7 °C for 1 min). A complete list of TaqMan probes are shown in Supplementary Table S1. In order to systematically compare all target genes and limit interpolated errors, the same threshold values of 0.2 were used for each gene. The data were then analysed using the cycling threshold (CT) relative quantification method, and genes with CT values < 35 were regarded as transcriptionally expressed [26]. A heatmap showing the gene expression pattern variance was built from reciprocals of CT values, using the ‘GenePattern’ web software (http://www.broadinstitute.org). Each colour patch in the heatmap represents the relative gene expression level, with a continuum of expression levels from dark blue (lowest) to bright red (highest).

### 2.3. P2X4R Agonist and Antagonists

P2X4R agonist adenosine 5′-triphosphate (ATP) disodium salt hydrate, competitive antagonist 5-(3-bromophenyl)-1,3-dihydro-2Hbenzofuro [3,2-e]-1,4-diazepin-2-one (5-BDBD), and allosteric antagonist 10-[(4-Methylphenyl)sulfonyl]-10H-phenoxazine (PSB-12062) were all purchased from Sigmal-Aldrich. 50 μM ATP was used in the calcium influx assay in order to maximally activate P2X4R but not P2X7R [27,28]. Both 5-BDBD and PSB-12062 powder were reconstituted in DMSO and then serial diluted with PBS into a stock concentration of 200 μM and 300 μM, respectively. The final working concentrations of 5-BDBD and PSB-12062 were 1.0 μM and 1.5 μM for all in vitro experiments, determined based on previously published IC_50_ values [28,29].

### 2.4. Calcium Influx Assay

Fluo-4 Direct™ (Thermo Fisher Scientific) was used to examine the calcium influx in PC3 cells treated with P2X4R specific antagonists (1.0 μM 5-BDBD or 1.5 μM PSB-12062) and agonist (50 μM ATP). Briefly, PC3 cells were seeded at 1 × 10^4^ cells per well in a 96-well plate and incubated for 24 h before being treated with P2X4R specific antagonists or vehicle in phenol-free DMEM containing 1× Fluoro-4-direct reagent for 45 min. The fluorescence (Excitation: 494 nm; Emission: 516 nm) was then read every 1.5 s over a 700 s period at 37 °C using the FlexStation 3 Multi-Mode Microplate Reader (Molecular Devices, CA, USA), while 50 μM ATP and 1 μM ionomycin (Sigmal-Aldrich) were automatically added at 30 s and 450 s, respectively. All readings were first normalized to the ionomycin-induced full calcium influx and then further normalized to the mean of the baseline reading (the mean value of the first 30 s) in order to generate a calcium influx curve over the 450-s period. The peak fluorescence intensity was then compared between the vehicle-, ATP-, and ATP plus antagonist-treated groups.

### 2.5. Viability Assay

The viability of PC3 and C4-2B4 cells treated with P2X4R antagonists was measured using an alamarBlue™ cell viability reagent (Thermo Fisher Scientific). In this assay, the PC3 and C4-2B4 cells were seeded at a density of 3 × 10^3^ cells/well in triplicates in 96-well plates at Day 0, in 5% FBS DMEM medium containing antagonists or vehicle. The alamarBlue™ cell viability reagent was added to each well at a dilution of 1:10 at 24, 48, 72, and 96 h post-seeding. After 3 h of incubation, the absorbance was measured in an EnSight Multimode Plate Reader (PerkinElmer, Waltham, MA, USA) with the excitation at 570 nm and emission at 600 nm.

### 2.6. Proliferation Assay

The proliferation of PC3 and C4-2B4 cells was measured by using the cellular DNA content and fluorescence-based CyQUANT™ NF Cell Proliferation Assays (Thermo Fisher Scientific). PC3 and C4-2B4 cells were seeded at 2 × 10^3^ cells/well in medium with antagonists or vehicle in 96-well plates. The treatment medium was replaced with 50 μL of 1× dye binding solution at 24, 48, 72, and 96 h post-seeding. After one hour of incubation, the fluorescence was measured in an EnSight Multimode Plate Reader (PerkinElmer) with the excitation at 485 nm and emission at 530 nm.

### 2.7. Apoptosis Assay

PC3 and C4-2B4 cells were seeded at 1 × 10^4^ cells per well in 96-well plates and cultured in medium with 1.0 μM 5-BDBD, 1.5 μM PSB-12062, 10 μM Doxorubucin (positive control), or the appropriate vehicle (DMSO in PBS) for 24 h. The cell apoptotic activity was then examined using the Cell Meter™ Caspase 3/7 Activity Apoptosis Assay (Stratech Scientific, Ely, UK), according to the manufacturer’s instructions. The fluorescence was measured using an EnSight Multimode Plate Reader (PerkinElmer) with the excitation at 490 nm and emission at 525 nm.

### 2.8. Migration Assay by Scratch Test

PC3 cells were seeded at a density of 2 × 10^5^ cells/well in 24-well plates for attachment overnight. The vertical scratches were produced by using 10-μL pipette tips, followed by gently washing the cells three times with serum-free medium to remove detached cells. 5 μg/mL Mitomycin C (Sigmal-Aldrich) was added to inhibit cell proliferation. After a 1-h incubation in Mitomycin C, cells were washed with serum free medium and replenished with medium containing 1 μM 5-BDBD, 1.5 μM PSB-12062, or vehicle controls. The cell migration was then monitored for 18 h with images taken at 0, 6, 12, and 18 h time points with a digital SPOT camera attached to an inverted Nikon phase contrast microscope (Nikon Inc., Tokyo, Japan) at a magnification of 20×. The percentage changes of scratch closure were assessed and calculated by using ImageJ software (https://imagej.nih.gov).

### 2.9. Cell Migration Assay by Transwell

As C4-2B4 monolayers were easily detached with physical manipulation, these cells were not suitable for the examination of migration using scratch assays. A Transwell method was therefore adopted to test the migration of C4-2B4 cells. C4-2B4 cells were seeded at 2 × 10^5^ cells per well in 200 μL serum-free medium containing 1 μM 5-BDBD, 1.5 μM PSB-12062, or vehicle on top of the Transwell^®^ cell culture inserts (6.5 mm with an 8-μm pore polycarbonate filter, Corning, New York, NY, USA), while 500 μL of 10% FBS DMEM was added to the lower chamber as the chemoattractant. After 16 h of incubation, the inserts were fixed in 100% ethanol for 5 min, stained in eosin for 1 min and haematoxylin for 5 min, air-dried and mounted on slides in Faramount. Finally, the slides were scanned using the Pannoramic 250 Digital Scanner (3D HISTECH, Budapest, Hungary), and the percentage areas covered by migrated cells were measured by using ImageJ software.

### 2.10. Invasion Assay

Transwell^®^ cell culture inserts (6.5 mm with an 8-μm pore polycarbonate filter, Corning) were first coated with 0.5 μg/mL Matrigel (Corning) in serum-free medium at 37 °C for 2 h. 5 × 10^4^ PC3 or C4-2B4 cells were then seeded into the top chamber with 200 μL serum-free medium containing antagonists (1.0 μM 5-BDBD or 1.5 μM PSB-12062) or vehicles, and were treated with 5 μg/mL Mitomycin C for 3 h. 500 μL of 10% FBS DMEM was added to the lower chamber as the chemoattractant. After 72 h of incubation, the inserts were fixated in 100% ethanol for 5 min, stained in eosin for 1 min and haematoxylin for 5 min, and then mounted on a slide. The slides were scanned using the Pannoramic 250 Digital Scanner (3D HISTECH) at a 1.2× magnification. The percentage areas covered by invaded cells were analysed by using ImageJ software.

### 2.11. In Vivo Study

Six-week old male BALB/cAnNCrl immunocompromised (athymic nude) mice (Charles River, Kent, UK) were used as a xenograft model of PCa. Mice were housed in a controlled environment in Optimice cages (Animal Care Systems, CO, USA) with a 12-h light/dark cycle at 22 °C with an ad libitum 2018 Teklad Global 18% protein rodent diet containing 1.01% calcium (Harlan Laboratories, Huntingdon, UK) and water. Fourteen mice were subcutaneously injected with 100 μL of PC3 cell suspension (5 × 10^6^ cells in 50% PBS+ 50% Matrigel) in the right flank. The mice were then randomized into two groups (seven mice per group), and daily intraperitoneal injections of vehicle (20 μL DMSO) or the P2X4R antagonist 5-BDBD at a dosage of 10 mg/kg, determined based on previously published in vivo usages (1–25mg/kg) [30,31,32], was started one day after tumour cell inoculation. Tumour sizes were measured by using vernier calipers twice a week, after which the tumour volumes were calculated as ellipsoids (Volume = πABC/6; A = length, B = width, C = height) until the animals were euthanized three weeks post-tumour cell inoculation. All procedures complied with the UK Animals (Scientific Procedures) Act 1986 and were reviewed and approved by the local Research Ethics Committees of the University of Sheffield under Home Office project licence 70/8799 (Sheffield, UK).

### 2.12. Histology

Tumours were dissected and fixed in 10% buffered formaldehyde, and were then embedded in paraffin wax. Sections were cut at a 3-µm thickness, mounted on slides, and stained in eosin and haematoxylin. The slides were scanned using the Pannoramic 250 Digital Scanner (3D HISTECH).

### 2.13. Retrospective Analysis of Clinical Datasets

The Gene & Expression resource of the National Centre for Biotechnology Information (NCBI), Gene Expression Omnibus (https://www.ncbi.nlm.nih.gov/geo/) [33] was used to conduct an extensive search of microarray and RNA-seq clinical data by searching for the keywords ‘prostate cancer’. The search result was further filtered for studies performed on humans and specifically on patient samples. Datasets from patient samples with the accession numbers GDS1439 [34], GDS1746 [35], and GDS3289 [36] were downloaded, and the expression of P2X4R was quantitatively compared between benign and malignant prostate tissue samples.

### 2.14. Statistical Analysis

All data are expressed as the mean ± SD. The statistical significance was tested for by using an unpaired Student’s *t*-test with or without Welsh’s correction and a one-way ANOVA with a post hoc Tukey test or Chi-square test as appropriate, using the Prism 7.03 software (GraphPad). *p* < 0.05 was considered to be significant.

## 3. Results

### 3.1. P2X4R is the Highest Expressed Functional P2 Receptor in PCa Cell Lines

**Figure 1:**
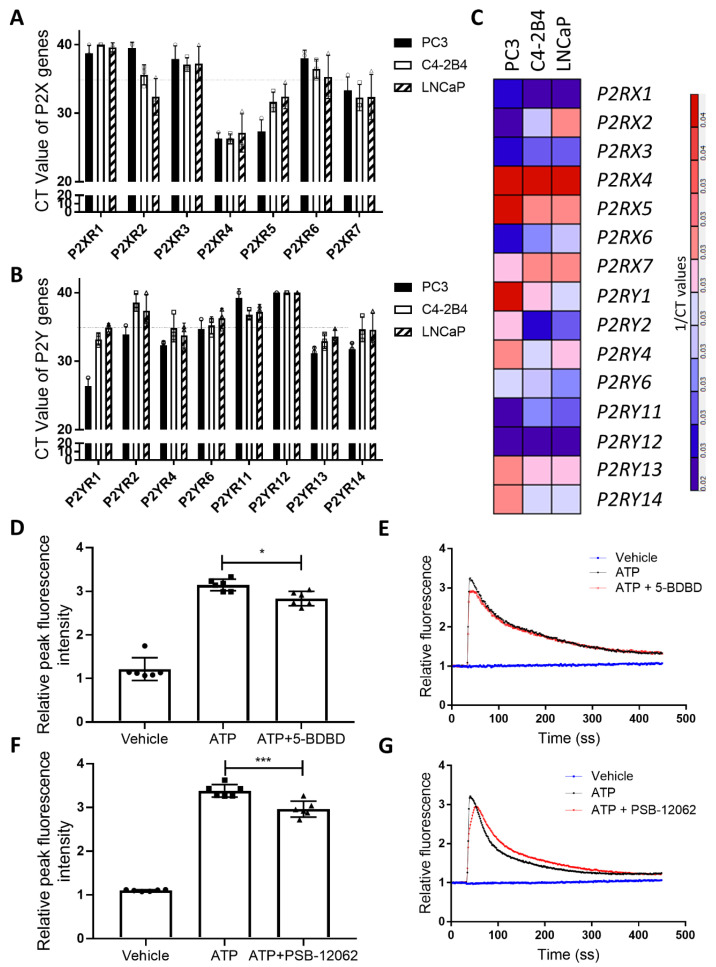
P2X4R is functionally expressed in PCa cell lines. Total mRNAs were isolated from three human PCa cell lines: PC3, LNCaP, and C4-2B4 cells. Individual quantitative RT-PCRs were performed to reveal the gene expression profile of (**A**) P2X and (**B**) P2Y receptors in these PCa cells. CT values < 35 were regarded as the transcription expression of P2X and P2Y receptors. (**C**) A heatmap showing the gene expression pattern variance was built from reciprocals of CT values, using the ‘GenePattern’ web software. Colour scale = 1/CT values. To understand whether P2X4R was functional, PC3 cells were pretreated with P2X4R specific antagonists, and then 50 μM ATP (to maximally activate P2X4R but not P2X7R)-induced calcium influx was examined using the Fluo-4 Direct™ agent. All data were normalized to both the baseline and ionomycin-induced full calcium influx. The peak fluorescence intensity was compared among the vehicle, ATP, and ATP plus (**D**) 1.0-μM 5-BDBD or (**F**) 1.5-μM PSB-12062. The representative ATP-induced calcium influx curve over a 450 s period with or without (**E**) 5-BDBD or (**G**) PSB-12062 antagonist pretreatments. Data are the mean ± SD, *n* = six biological repeats, one-way ANOVA with post hoc Tukey test, * *p* < 0.05, *** *p* < 0.001.

To understand the transcriptional expression pattern of P2 receptors in PCa cells, we performed individual quantitative RT-PCR on total mRNA isolated from three PCa cell lines: PC3, LNCaP, and C4-2B4. The results show that the RNA (CT value < 35) of P2X4, X5, X7, Y_1_, Y_4_, Y_13_, and Y_14_ receptors are expressed in all three cell lines (Figure 1A,B). Among these P2 receptors, *P2RX4* is the most highly expressed (CT value ~24), while the *P2RY14* receptor is the least expressed (CT value ~32) (Figure 1C). To further examine whether P2X4R is functional in PCa cells, we tested the ATP-induced calcium influx changes under the influence of the P2X4R specific antagonists 5-BDBD and PSB-12062. At the IC_50_ level (5-BDBD: 1.0 μM and PSB-12062: 1.5 μM), both antagonists are able to reduce the ATP-induced peak calcium influx by ~10% (Figure 1D–G), suggesting that P2X4R is functional in PC3 cells. However, we were unable to detect a calcium influx in C4-2B4 cells under the stimulation of extracellular ATP at a range of concentrations (50–200 μM) (Supplementary Figure S1A,B), suggesting the presence of non-typical P2X4R in C4-2B4 cells.

### 3.2. Inhibiting P2X4R Impairs Proliferation and Viability of PCa Cells but Not Apoptosis

**Figure 2:**
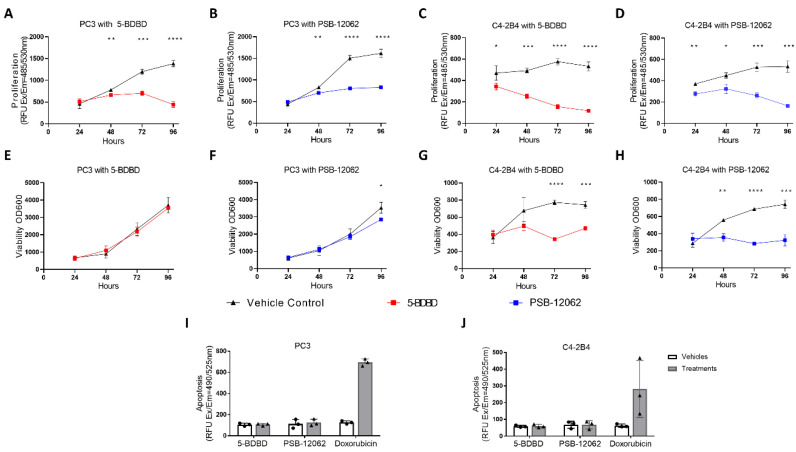
Effects of P2X4R inhibition on PCa proliferation, viability, and apoptosis. The effects of inhibiting P2X4R with specific antagonists (1.0 μM 5-BDBD or 1.5 μM PSB-12062) on the proliferation and viability of PCa cells (PC3 and C4-2B4) up to 96 h were examined by using the (**A**–**D**) CyQUANT^®^ cell proliferation assay and (**E**–**H**) alamarBlue™ cell viability assay. (**I**,**J**) The apoptotic activity after a 24-h treatment of antagonists or doxorubicin (as a positive control) was measured using the Cell Meter™ Caspase 3/7 apoptosis assay. Data are the mean ± SD, *n* = three biological repeats, Student’s *t*-test, * *p* < 0.05, ** *p* < 0.01, *** *p* < 0.001, **** *p* < 0.0001.

To determine whether P2X4R is involved in PCa cell biology, we first examined the changes in the proliferation, viability, and apoptosis of PCa cells with or without P2X4R inhibition. Using the DNA content-based CyQUANT^®^ cell proliferation assay, treatments with both 5-BDBD and PSB-12062 for up to 96 h significantly reduced the proliferation by >50% in PC3 cells (Figure 2A,B) and by >70% in C4-2B4 cells (Figure 2C,D). Next, using the cell metabolism activity-based alamarBlue™ assay, the viabilities of PC3 and C4-2B4 cells were examined over a 96-h period. Treatment with both antagonists significantly reduced the viability of C4-2B4 cells (36.8% and 56.4% reductions at 96 h in the 5-BDBD and PSB-12062 groups, respectively, *p* < 0.001, Figure 2G,H), while only PSB-12062 reduced the viability of PC3 by <20% at 96 h (Figure 2E,F). The caspase 3/7 activity apoptosis assay demonstrated that inhibiting P2X4R with both antagonists for 24 h did not affect the apoptosis of either the PC3 cells (*p* > 0.05, Figure 2I) or C4-2B4 cells (*p* > 0.05, Figure 2J). Significant levels of apoptosis were induced by the treatment of the cells with the cytotoxic agent doxorubicin (Figure 2I,J).

### 3.3. Inhibiting P2X4R Reduces PCa Cell Mobility

**Figure 3:**
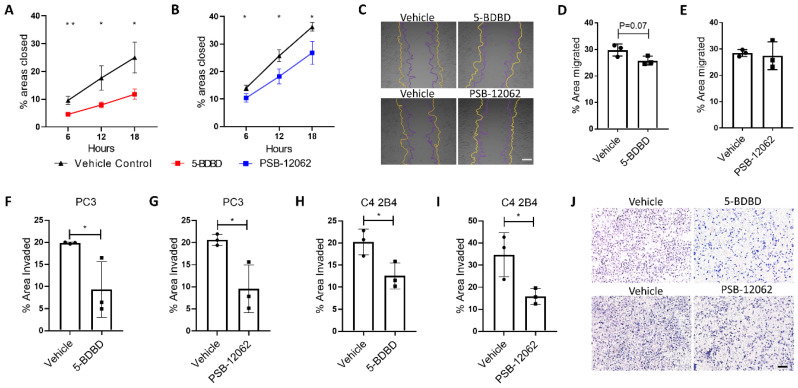
Inhibiting P2X4R with antagonists impairs PCa cell mobility. Changes in the migration and invasiveness of PC3 and C4-2B4 cells in response to the 1.0 μM 5-BDBD or 1.5 μM PSB-12062 treatment were examined using a scratch test and Transwell assays, respectively. For the scratch test migration assay, percentage areas closed after scratch were compared at 6, 12, and 18 h between (**A**) 5-BDBD- or (**B**) PSB-12062-treated and vehicle groups. (**C**) A representative image of the scratch test assay. Scale bar = 100 μm. For C4-2B4 cells, a Transwell migration assay was adopted with 10% FBS DMEM as the chemoattractant in the lower chamber. Percentage areas that migrated within 16 h were measured and compared between (**D**) 5-BDBD- or (**E**) PSB-12062-treated groups and the appropriate vehicle groups. (**F**–**I**) The invasion assay was performed by examining the ability of PC3 and C4-2B4 cells invading Matrigel-coated Transwell inserts over a 72-h period and comparing antagonist and vehicle groups. (**J**) A representative comparison image of the PC3 cells invading Matrigel-coated Transwell inserts, with or without P2X4R antagonist treatments. Scale bar = 200 μm. Data are the mean ± SD, *n* = three biological repeats, Student’s t-test, * *p* < 0.05, ** *p* < 0.01.

In the scratch test migration assay, PC3 cells treated with 5-BDBD showed a significant ~50% reduction in their migration ability at all time points up to 18 h when compared to the vehicle control group (*p* > 0.05, Figure 3A,C). Treatment with PSB-12062 caused significant, slightly lower (25–30%) reductions in PC3 migration over the 18-h time course (Figure 3B,C). In the Transwell cell migration assay, 16 h of 5-BDBD treatment showed a 13% reduction in migration (*p* = 0.07, Figure 3D), while PSB-12062 did not affect the migration of C4-2B4 cells (Figure 3E). In the invasion assay, treatment with both antagonists for 72 h significantly reduced the ability of both PC3 and C4-2B4 cells to invade Matrigel-coated Transwell inserts when compared to vehicle controls (~50% reduction, Figure 3F–J).

### 3.4. P2X4R Antagonist Shows Anti-Tumourigenic Effects in a PCa Xenograft Model

**Figure 4:**
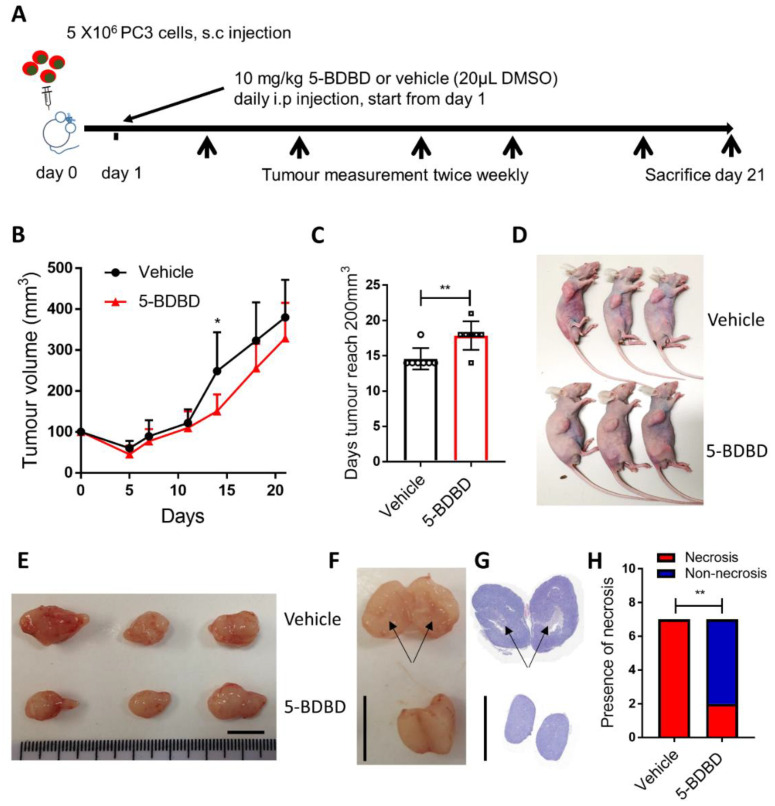
P2X4R antagonist 5-BDBD delays the PCa growth in vivo. (**A**) Schematic outline of the in vivo study. Briefly, six-week old BALB/cAnNCrl immunocompromised mice were subcutaneously (s.c) injected with 5 × 10^6^ PC3 cell suspension with 50% PBS + 50% Matrigel. After randomization, the mice were daily intraperitoneally (i.p) injected with the P2X4R antagonist 5-BDBD at a dosage of 10 mg/kg or vehicle (20 μL DMSO), starting from day one until being euthanized three weeks post-tumour cell inoculation. (**B**) Tumour sizes were measured by using vernier calipers twice a week, and then tumour volumes were calculated as ellipsoids and compared between treated and vehicle groups. (**C**) Days needed for tumours to reach 200 mm^3^ were also compared between treated and vehicle groups. Data are the mean ± SD, *n* = 7, Student’s t-test, * *p* < 0.05, ** *p* < 0.01. Representative images of (**D**) tumour-bearing mice and (**E**) dissected tumours, comparing treated and vehicle groups at endpoint. (**F**) Dissected tumours were then cut in half and revealed a necrotic centre in the tumour from the vehicle control group but not the treated group. (**G**) This was further confirmed by H&E staining of the tumours. Scale bar = 1 cm. (**H**) A statistical analysis with a Chi-square test suggested a significantly reduced presence of necrosis in the 5-BDBD-treated group compared to the vehicle. *n* = 7, ** *p* < 0.01.

To understand whether inhibiting P2X4R will affect the progression of PCa in vivo, we performed a xenograft study. The growth of PC3 cells subcutaneously injected into BALB/c nude mice, followed by the daily administration of 10 mg/kg 5-BDBD or vehicle (DMSO), was followed for three weeks (Figure 4A). The 5-BDBD treatment significantly reduced the growth of the PC3 tumour by day 14 (39.4% reduction, *p* = 0.0278, Figure 4B) and led to a significant 3.3-day delay until tumours reached the 200-mm^3^ volume that was double the initial seeding volume (14.6 days in the control vs. 17.9 days in the 5-BDBD treated group, *p* = 0.005, Figure 4C). However, although the significant reduction in tumour volume had been lost by day 21, a 13.3% reduction in the tumour volume remained in the treatment group, compared to the vehicle at the end point (Figure 4B,D,E). Further examination of dissected tumours by gross observation and histology showed that tumours from vehicle groups clearly contained areas of necrosis (marked by black arrows, Figure 4F,G), while there was a significantly reduced tumour necrosis in the treated group (two in seven in the 5-BDBD-treated group vs. seven out of seven in the vehicle group, *p* = 0.0053, Chi-square test, Figure 4H).

### 3.5. Retrospective Analysis of Clinical Datasets Suggests That P2X4R Associates with PCa Malignancy

**Figure 5:**
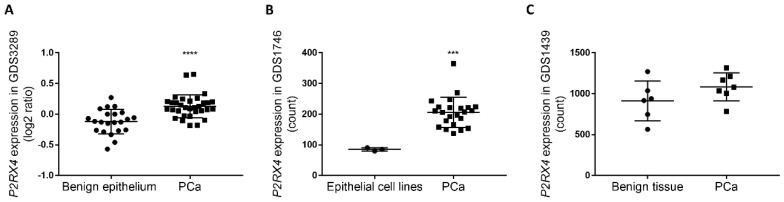
Clinical data suggests an association between *P2RX4* expression and PCa malignancy. Clinical datasets with accession numbers GDS1439 [34], GDS1746 [35], and GDS3289 [36] were downloaded from Gene Expression Omnibus (https://www.ncbi.nlm.nih.gov/geo/) [33]. The expression of the *P2RX4* gene was quantitatively compared between primary PCa samples and (**A**) benign prostate epithelium tissues (GDS3289, PCa samples, *n* = 32; benign prostate epithelium, *n* = 22), (**B**) primary human prostate epithelial cell lines (GDS1746, PCa samples, *n* = 23; human prostate epithelial cell lines, *n* = 3), and (**C**) benign prostate tissues (GDS1439, PCa samples, *n* = 7 from five individual patients and two patient tissue pools; benign prostate tissues, *n* = 6). Student t-test, *** *p* < 0.001, **** *p* < 0.0001.

To understand whether the expression of *P2RX4* was associated with PCa progression in clinical settings, we searched the Gene Expression Omnibus database and identified three clinical datasets, GDS1439 [34], GDS1746 [35], and GDS3289 [36], which characterized the transcriptomic profile in PCa samples and benign prostate tissues. In the two datasets GDS1746 and GDS3289, the expression of the *P2RX4* gene was significantly upregulated in the PCa samples when compared to benign prostate epithelium tissues (*p* < 0.0001, GDS3289, Figure 5A) or primary human prostate epithelial cell lines (*p* = 0.0003, GDS1746, Figure 5B). In the other dataset GDS1439 with a smaller cohort of clinically localised primary PCa samples, *P2RX4* gene expression was upregulated when compared to benign prostate tissues (18.9% increase, *p* = 0.1612, Figure 5C).

## 4. Discussion

In this study, we performed a series of in vitro and in vivo assays using PCa models and combined the generated data with a retrospective analysis of clinical datasets in order to determine the role of P2X4R in PCa growth. The qRT-PCR results clearly demonstrate that P2X4R is the most highly expressed P2 receptor at the transcriptional level across the examined human PCa cell lines, which is consistent with expression profiles in other tissues or organs [9,10]. We demonstrated that two P2X4R selective antagonists (5-BDBD and PSB-12062) could both significantly reduce the ATP-induced calcium influx in PC3 cells, indicating that P2X4R was functional in PC3 cells and also that these two antagonists could be used for subsequent tumour biology studies to determine the activities of P2X4R. Interestingly, extracellular ATP-induced calcium influxes in C4-2B4 cells were not detected, suggesting the possibility of a non-typical P2X4R in these cells; splice variants of the human P2X4R are known to lack a typical ATP-evoked channel activity [37,38]. Inhibiting P2X4R with 5-BDBD and PSB-12062 significantly decreased the proliferation and viability but did not affect the apoptosis of PCa cells in vitro. The antigrowth effects of inhibiting P2X4R were consistent with previous evidence in glioblastoma multiforme (GBM), where P2X4R was shown to regulate cell growth via the brain-derived neurotrophic factor (BDNF)/Tropomyosin receptor kinase B (TrkB)/Activating Transcription Factor 4 (ATF4) signalling pathway [20,39]. However, the lack of enhanced apoptosis in PCa cells treated with antagonists was a little surprising, as P2X4R has also been suggested to have an anti-apoptotic role in GBM [20]. This could be because inhibiting P2X4R alone is not sufficient enough to affect PCa apoptosis, as has been shown for breast cancer, where P2X4R modulates cell death via coordinating with other receptors/signalling such as the P2X7R and Pannexin-1 channel [40]. Furthermore, consistent with findings by Ghalali et al. [25], our migration and invasion data demonstrates that inhibiting P2X4R impairs the mobility of PCa cells. All these results suggest that P2X4R plays a comprehensive role in multiple aspects of PCa biology, which warrants further studies to reveal the molecular and cellular mechanisms involved. One of the potential candidate pathways mediating these effects could lie downstream of P2X4R Ca^2+^ influx-induced p38 MAPK phosphorylation [11,14]. p38 MAPK has been shown to be associated with cell proliferation in tumours from patients and PCa cell lines [41,42]. p38 MAPK also plays a dual role in cell death regulation in solid tumours, depending on the type of stimulus and cells [43]. In PCa, active p38 promotes cellular apoptosis, but this needs to coordinate with other signals (e.g., the PI3K/Akt pathway) to determine cellular outcomes [43,44]. Additional studies revealed the pro-oncogenic role of p38 in promoting PCa migration and invasion [45,46]. Based on this evidence, one can speculate that the inactivation of the p38 MAPK in PCa will resemble our in vitro observations, and further investigations are therefore warranted to confirm the involvement of this mechanism.

Our in vivo data provides further evidence that P2X4R is involved in PCa tumour formation. With the daily treatment of the selective P2X4R antagonist 5-BDBD, mice bearing subcutaneously inoculated PC3 cells had a significantly reduced tumour mass by two weeks postinjection when compared to vehicle control mice. However, differences started to diminish during the final week of the experiment. This is possibly due to the fact that the tumours in the control group are necrotic after two weeks, as a result of which their growth is reduced. Overall, these data indicate that inhibiting P2X4R in vivo delays the initiation and early growth of PCa tumours rather than causing cell death. This is consistent with our in vitro data that shows that inhibiting P2X4R in PCa cells impairs proliferation but does not induce apoptosis. More specifically related to the PC3 cells used in this xenograft study, our data indeed showed that 5-BDBD did not affect the viability of PC3 cells, the growth inhibitory effects being more likely to be confined to limiting DNA synthesis and proliferation through p38 MAPK inhibition [42]. Further in vivo studies can be done in order to depict a full picture of the role of P2X4R in PCa progression, using different cell lines and antagonists as well as alternative in vivo models. For example, with a pro-mobility role suggested by our in vitro data, the involvement of P2X4R in PCa metastasis (e.g., PCa bone metastases) should be explored in in vivo models (e.g., via intracardiac inoculation route). A combination treatment study with the P2X4R antagonist and cytotoxic drugs (e.g., Docetaxel) would reveal whether better anti-PCa outcomes could be achieved. Furthermore, syngeneic models (e.g., a murine RM1 model) should be used in order to take into consideration an intact immune system.

Our in vivo study also showed that inhibiting P2X4R reduced tumour necrosis. This may be a result of the slower initiation/early growth of tumours in treated animals. In PCa tumours, necrosis is associated with a poor prognosis [47,48]. Mechanistically, evidence has suggested that ATP release from the P2X4R/P2X7R-gated Pannexin-1 channels can drive cell death via a mixed apoptotic and necrotic mode [40], which could be the reason why tumour necrosis was largely absent in the 5-BDBD-treated mice. Other mechanisms, such as the involvement of P2X4R in the inflammatory response, could also contribute to the effects observed in the presence of the inhibitor. The expression of P2X4R has been shown to be co-localised with the pro-inflammatory cytokine IL-1β in renal tubule epithelial cells [49,50], while a prolonged exposure to IL-1β induces necrosis in various cell types including tumour cells [50,51,52], although others have argued that IL-1β is more involved in inducing apoptotic cell death [53].

Through a retrospective analysis of multiple publicly available clinical datasets, we have shown that the transcriptional expression of *P2RX4* is significantly upregulated in patient PCa samples when compared to benign prostate tissues or cells, suggesting a potential involvement of P2X4R in the development and growth of PCa malignancies. This observation, in combination with our studies using PCa models, suggests that P2X4R is a promising new target for the development of PCa treatments. However, since P2X4R is widely expressed by multiple cell/tissue types and involved in many biological functions (e.g., it plays a key role in pain), one should be cautious when clinically targeting P2X4R in order to avoid serious side effects. Others have shown some adverse phenotypes in P2X4-deficient mice, such as a risk of hypertension [54], cell senescence in the hippocampus [55], and an increased voluntary ethanol intake in mice [56]. These adverse effects could be from the interruptions of P2X4R-closely linked genes, such as the P2X7R gene, due to the neomycin cassette inserted in the P2X4 locus when the knockout mouse lines were created [10]. Therefore, future research would need to elucidate the coordination between P2X4R and other receptors/signalling, improving pharmaceutical safety and efficacy and applying cell/tumour-targeted drug-delivery systems in order to achieve the clinical benefits of novel P2X4R-targeted, PCa treatment strategies.

In conclusion, the involvement of P2X4R in PCa progression was comprehensively examined for the first time using a series of in vitro and in vivo assays, combined with a retrospective analysis of clinical datasets. The results support our original hypothesis that P2X4R enhances tumour formation and growth in PCa and is targetable using already available inhibitors.

## Supplementary Materials

The following are available online at https://www.mdpi.com/2073-4409/9/11/2511/s1, Figure S1: ATP induced calcium influx can not be detected in C4-2B4 cells., Table S1: List of TaqMan qRT-PCR gene expression assays.
