# Supplementary material for: Inhibiting the P2X4 Receptor Suppresses Prostate Cancer Growth In Vitro and In Vivo, Suggesting a Potential Clinical Target
Source: Cells. 2020 Nov 20;9(11):2511. doi: 10.3390/cells9112511 (PMC7699771; doi:10.3390/cells9112511)
Supplement: Supplementary file 1 [file cells-09-02511-s001.pdf]

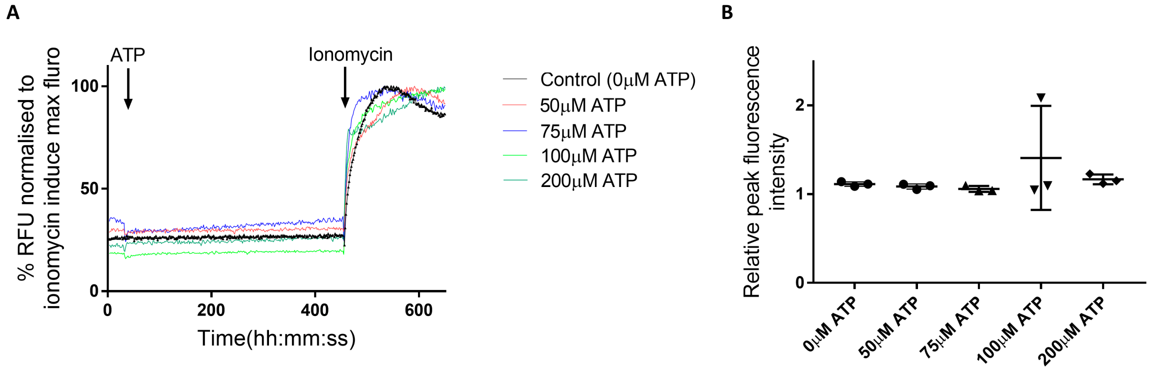

Supplementary Figure S1. ATP induced calcium influx can not be detected in C4-2B4 cells. The ATP (0-200 μM) induced calcium influx in C4-2B4 cells was examined using the Fluo-4 Direct™ agent. (A) Representative ATP induced calcium influx curve over a 650-seconds period (ATP was added at 30 sec and ionomycin was added at 450 sec). RFU was normalized to the maximum fluorescence induced by ionomycin. (B) Peak fluorescence intensities were normalized to both baseline and ionomycin induced full calcium influx, and then compared among vehicle and different dosages of ATP groups. n=3 independent biological repeats.

Supplementary Table S1. List of TaqMan qRT-PCR gene expression assays.

Gene	Assay ID
P2RX1	Hs00175686_m1
P2RX2	Hs04176268_g1
P2RX3	Hs01125554_m1
P2RX4	Hs00602442_m1
P2RX5	Hs01112471_m1
P2RX6	Hs01003997_m1
P2RX7	Hs00175721_m1
P2RY1	Hs00704965_s1
P2RY2	Hs00602525_m1
P2RY4	Hs00267404_m1
P2RY6	Hs00366312_m1
P2RY11	Hs01038858_m1
P2RY12	Hs00224470_m1
P2RY13	Hs03043902_s1
P2RY14	Hs01848195_s1
